# HPV - immune response to infection and vaccination

**DOI:** 10.1186/1750-9378-5-19

**Published:** 2010-10-20

**Authors:** Margaret Stanley

**Affiliations:** 1 Department of Pathology, University of Cambridge, Tennis Court Road, Cambridge CB2 1QP, UK

## Abstract

HPV infection in the genital tract is common in young sexually active individuals, the majority of whom clear the infection without overt clinical disease. However most of those who develop benign lesions eventually mount an effective cell mediated immune (CMI) response and the lesions regress.

Failure to develop effective CMI to clear or control infection results in persistent infection and, in the case of the oncogenic HPVs, an increased probability of progression to CIN3 and invasive carcinoma. The prolonged duration of infection associated with HPV seems to be associated with effective evasion of innate immunity thus delaying the activation of adaptive immunity.

Natural infections in animals show that neutralising antibody to the virus coat protein L1 is protective suggesting that this would be an effective prophylactic vaccine strategy. The current prophylactic HPV VLP vaccines are delivered i.m. circumventing the intra-epithelial immune evasion strategies. These vaccines generate high levels of antibody and both serological and B cell memory as evidenced by persistence of antibody and robust recall responses. However there is no immune correlate - no antibody level that correlates with protection. Recent data on how HPV infects basal epithelial cells and how antibody can prevent this provides a mechanistic explanation for the effectiveness of HPV VLP vaccines.

## Introduction

It is chastening to remember that until the mid to late 1970's - 40 years ago - infection with human papillomaviruses was regarded as trivial. The received wisdom was that HPV caused warts, benign epithelial proliferations of cutaneous and internal squamous mucosal surfaces and nothing more. This view was overset by two quite disparate reports - one clinical and one, fundamental molecular virology. In 1976 Meisels & Fortin published a report in which they showed that the "halo cell" in pap smears read by the cytologist was a koilocyte - the pathognomic cell of HPV infection [1]and that low grade or mild dysplasia of the cervix had the histological features of a papillomavirus infection [2]. Shortly thereafter Lutz Gissmann in the zur Hausen laboratory in Erlangen cloned from genital warts 'condyloma acuminata', a novel HPV DNA classified as HPV6 [3]. Using the DNA sequences from this as a probe Lutz Gissmann then partially cloned from genital warts another new HPV - HPV11 [4]. HPV11 DNA was used by members of the zur Hausen laboratory as a probe and in 1983 they reported the cloning of a novel HPV from cervical carcinoma biopsies - HPV16 [5], followed a year later by another new HPV in cervical carcinoma - HPV18 [6].

### Burden of Disease: high risk or oncogenic HPV strains

Developments since then need little repeating - infection with one of a subset of mucosal HPVs is the major risk factor for the development of cervical cancer. There are at least 15 oncogenic (cancer causing) HPV types or strains but HPV16 and 18 are the most virulent causing an average 70% of all cervical cancers [7]. The spectrum of HPV associated cancers now extends with HPV the major aetiological agent in squamous cell carcinoma of the anus, tonsil and base of tongue and a significant contributor to squamous cell carcinoma of the vulva, vagina, penis, larynx and head and neck http://globocan.iarc.fr/ Overall it is estimated that HPV is the causal agent in 5% of all human cancers with HPV16 by far and away the major player.

### Burden of disease: low risk HPV strains

The contribution to the cancer burden is very significant but the disease burden of the "benign" or low risk HPVs - mainly 6 and 11 - should not be underestimated. Genital warts - condyloma acuminata - are not a preferred topic of polite conversation but they are the commonest viral sexually transmitted infection with a life time risk of acquisition of 10% [8]. This represents a huge disease burden and major health economic cost in developed countries. HPV infection in the larynx resulting in recurrent respiratory papillomatosis is a rare but devastating disease. The juvenile form of this disease - 4/100,000 live births - results in young infants and children with extensive warts in the larynx [9]. These lesions have to be surgically removed to prevent airway occlusion and these unfortunate children experience repeated surgical interventions and the accompanying anaesthesia to remove the warts. A small fraction of affected individuals have bronchial and pulmonary extension that results in mortality [10].

### Natural history of HPV infection

HPV infection is determined by detection of DNA, not isolation of virus. Genital HPV infection is predominantly, but not exclusively, a sexually transmitted infection but penetrative vaginal or anal sexual intercourse is not a prerequisite for infection, virus is transmitted by skin to skin contact via intimate contacts of the genitalia or other mucosal surfaces [11]. These infections are very common and it is estimated that 50-80% of sexually active men and women will acquire a genital HPV (both high and low risk) in their lives. The peak period of acquisition is soon after the start of sexual activity [12] and the risk of infection increases with the number of sexual partners [13].

**Figure 1:**
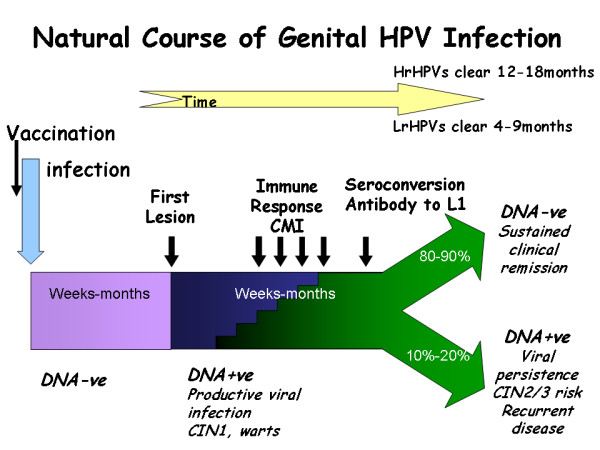
**Mechanisms of HPV infection in women genital tract**.

The natural history of genital HPV infection is illustrated in Figure 1. HPV is highly infectious with an incubation period ranging from 3-4 weeks to months or years, the duration of this latent period probably relating to the dose of virus received. Eventually for reasons that are still not understood permissive viral growth commences, viral DNA can be detected and infectious virus is shed. This phase of active replication also persists for a variable length of time but eventually the vast majority of infected individuals mount an effective immune response becoming DNA negative with subsequent sustained clinical remission from disease [14]. Effective immunity consists of a cell mediated response to the early proteins, principally E2 and E6 [15], necessary for lesion regression accompanied or followed by sero-conversion and antibody to the major capsid protein L1.

A minority variously estimated as being between 10-20% do not effectively clear virus, they remain DNA positive with a persistent active viral infection and it is these individuals who are at risk for progression to high grade precancers in the cervix - cervical intra-epithelial neoplasia CIN2/3 and thus invasive cancer [11]. HPV infections are exclusively intra-epithelial; virus infects basal epithelial cells probably at low copy number and amplifies in a first round of DNA amplification to 100 or so nuclear episomes per cell. Virus remains in an episome maintenance phase with minimal viral gene expression in the proliferating compartment of the epithelium. Viral gene expression becomes maximal in the differentiating non cycling cells of the upper 1/3 - 2/3 of the epithelium with viral capsid synthesis, assembly and shredding confined to the superficial differentiating squames [16]. This replication strategy with tight control over early gene expression ensures that in dividing cells the powerful oncogenic properties of the E6/E7 proteins of the high risk HPVs are not allowed to flourish. However, if viral infection becomes persistent then the probability of molecular accidents deregulating control of E6/E7 oncogene expression in mitotically active cells will increase [17] and neoplastic progression starts.

## HPV immune evasion strategies

Clearly HPV effectively evades recognition by the immune system for many months so how does this occur? The infectious cycle itself is a mechanism for this - there is no viraemia, very low levels of viral protein are expressed but crucially HPV is not cytolytic. Virus replication and assembly occur in cells already destined for death by anoikis or "death by natural causes", there is no inflammation and no danger signal to alert the immune system. The interferon response for HPV infection, a key antiviral defence mechanism [18], is actively suppressed with the E6 and E7 proteins of the high risk HPVs inhibiting the interferon receptor signalling pathways and the activation of the interferon response genes [19]. The E7 proteins downregulate TLR9 [20] and overall HPV effectively evades the innate immune response delaying the activation of adaptive immunity.

## Prophylactic HPV Vaccines

### Humoral response in natural HPV infections

The most effective control of viral infections is prophylactic vaccination and the development of prophylactic HPV vaccines is one of the scientific triumphs of the past 20 years. Sero conversion in natural genital infection results in detectable serum neutralising antibody to the major capsid protein L1 but this response is slow with an average time to sero-conversion after the first detection of HPV16 DNA of 8-9 months, antibody concentrations are low and only 50-70% of women with incident HPV infection sero convert [21]. However, anti L1 antibody persists in many women for at least 10 years [22] and these low levels of antibody are protective against disease [23]. Neutralising anti L1 antibody was known to be protective against high dose viral challenge in the classic animal models of papillomavirus infection, the dog, cow and rabbit, supporting the notion that a prophylactic HPV vaccine would be effective. Generating such vaccines was not a trivial issue; papillomaviruses cannot be grown in bulk in tissue culture so traditional live attenuated or killed vaccines were not feasible. Expression of the soluble native L1 protein via prokaryotic vectors was generally unsuccessful however, seminal technical advances in the early 1990's showed that if the L1 protein was expressed via eukaryotic expression systems, assembly into virus like particles occurred [24,25]. These L1 VLPs were morphologically and antigenically comparable to wild type virus and neutralising antibody generated by immunisation with L1 VLP protected against viral challenge in animal systems [26,27].

### HPV L1 VLP vaccines

There are two licensed HPV L1 VLP prophylactic vaccines - Cervarix^® ^a bivalent HPV 16/18 vaccine from GlaxoSmithKline Biologicals (GSK) and Gardasil^®^, a quadrivalent HPV16/18/6/11 vaccine from MSD Merck. Both vaccines have been shown in randomised control trials to be highly efficacious against HPV 16/18 caused high grade cervical intra epithelial disease (CIN2/3) in 15-26 year old women naïve for these HPV types at trial entry and during the 3 shot immunisation schedule (0, 1 or 2 and 6 months) [28,29]. Additional trial endpoints were evaluated for the quadrivalent vaccine and high (> 96%) efficacy was shown for HPV6/11/16/18 caused vulvar and vaginal intra epithelial neoplasia and external genital warts [30]. In RCTs in 16-23 year old men the quadrivalent vaccine has been shown to achieve >90% efficacy against HPV6/11/16/18 caused external genital warts in heterosexual men and > 73% efficacy against anal intra-epithelial neoplasia in homosexual men (Palefsky J personal communication 2010).

There is preliminary evidence for population effectiveness for the quadrivalent vaccine. In 2008 Australia offered publicly funded immunisation with the quadrivalent vaccine to all women aged 12-26 years and achieved, overall, 60-70% coverage. Audit of new female patients with external genital warts, 28 years or less attending a large sexual health clinic in Melbourne, Australia before and after vaccine introduction showed a fall in incidence of EGW after vaccine introduction in this age group of up to 40% Women older than 28 years who were not in the group eligible for free vaccines showed no fall in incidence [31].

## Mechanisms of HPV vaccine protection

### VLP immunogenicity

The current assumption is that the protection afforded via these vaccines is antibody mediated since passive immunisation is protective in animal models [26,27]. HPV L1 VLP vaccines are highly immunogenic generating antibody concentrations after the 3^rd ^immunisation that are 1-4 logs higher than those in natural infections [32,33]. In published studies serum neutralising antibody persists with geometric mean titres (GMTs) about 1 log greater than natural infection for the 7-9 year duration of the published studies, [34,35]. Mathematical modelling predicts slow decay of antibody over a 30-50 year period and potentially, therefore, protection over that time [36,37]. Both type specific and cross neutralising antibodies are generated by VLP vaccines although concentrations of cross neutralising species are on average 1-2 logs lower than type specific [38]. At the present there is no immune correlate, no antibody concentration (or other immune measurement) has been defined that correlates with protection. However other than information on antibody concentration (both IgG and neutralising) there is little published data on other parameters such antibody affinity and avidity maturation that may be relevant in this context. Early reports indicate that avidity maturation does not correlate with antibody concentration or circulating memory B cells [39].

### Route of immunisation

The enhanced immunogenicity of VLP immunisation in contrast to natural infection very probably relates to the route of immunisation. Natural infection is exclusively intra epithelial, virus is shed from mucosal surfaces and there is little or no viraemia. Viral antigen therefore poorly accesses the lymphatics and draining lymph nodes (DLN) where immune responses are initiated. Furthermore the most likely antigen presenting cells (APC) are macrophages and Langerhans cells both of which will be relatively ineffective in the non-inflammatory environment of a productive HPV infection (HPV is not cytolytic). VLP vaccines in contrast are delivered by injection via the intra-muscular route with immediate access to the vasculature and lymphatics. They rapidly encounter stromal dendritic cells that, in the inflammatory milieu induced by vaccination, are strongly activated to mature and migrate to the DLN initiating helper T cell responses. At the same time VLPs either surface bound to APC or other local immunocytes are shuttled into the lymph node encountering and priming naïve B cells in the follicle and the whole cascade of events that will result in protective immunity is started. Furthermore the VLP is intrinsically very immunogenic. The exquisite repeat pattern of L1 capsomers that stud the particle powerfully activate the innate immune sensors [40,41], even in the absence of adjuvant [42], kick starting the adaptive immune response. These crucial early events set the pattern for the subsequent immune cascades.

### Immune memory

The antibody response after the 3 dose immunisation schedule follows the expected pattern. After each vaccine dose antibody levels increase until a peak antibody concentration is achieved one month after the 3^rd ^and final dose in the primary schedule. Antibody concentrations wane over the subsequent 12 - 18 months but then stabilise at a plateau level with GMTs on average 10 times greater than in the placebo groups [32,33]. This pattern is consistent with the notion of the generation of a large population of antibody secreting plasma cells after dose 3, with varying life spans but some with the ability to migrate to the bone marrow surviving as long lived plasma cells maintaining a low but constant antibody production [43]. Antigen challenge at 60 months post dose 1 with the quadrivalent vaccine results in a rapid and robust anamnestic response with antibody levels rising within 3-5 days to levels greater than that achieved at peak in the initial immunisation schedule demonstrating the presence of reactive memory B cells [44]. A similar pattern of antibody response is shown by the bivalent vaccine and although there is no published data on a recall response, antigen specific circulating memory cells can be detected after each vaccine dose peaking after the third immunisation [45]. Collectively these data strongly imply that HPV VLP immunisation generates both components of the antibody memory response ie serological memory and reactive memory [46], a prerequisite for long term vaccine induced protection.

### Mechanism of virus entry

HPVs have an exclusively intra-epithelial life cycle with either minimal or no viraemia so how can serum neutralising antibody protect against infection? One explanation often offered is that transudated serum antibody in cervical secretions is the major mechanism of protection. This must certainly contribute but cannot explain protection at the well keratinised and comparatively dry surfaces of the vulva and vagina, penis and peri-anal skin. Virus neutralising antibody prevents virus entry into cells thus the questions to be addressed are how does HPV access and infect the basal cell of stratified squamous epithelium and how do neutralising antibodies prevent this. The emerging evidence is that HPV cell entry is a complex process involving epithelial microtrauma and wound repair [47]. HPV infects basal epithelial cells via micro wounds or microabrasions that remove the full thickness epithelium, but retain the basement membrane [48]. The virus binds via L1 first to the basement membrane and then to the cellular receptor on the migrating wound keratinocyte [49]. L1 antibodies can block both of these interactions and those antibodies that block basement membrane binding neutralise at extremely low concentrations [50]. This process of virus entry is slow and current estimates are that there is a minimum of 12-14 hours before HPV enters the wound keratinocyte [47] and, since the microwound will be accompanied by a serous exudate, exposure to serum antibody will be rapid.

## Summary and Conclusions

Infection with an oncogenic HPV particularly HPV 16 is associated with 5% of all human cancers but predominantly cervical cancer and other ano-genital cancers. HPV infection with both oncogenic and non oncogenic types is common in young, sexually active men and women but the majority of infections resolve due to a cell mediated response to early non structural proteins. This response is usually accompanied by sero-conversion with type specific neutralising antibody to the major capsid protein L1.

Prophylactic HPV vaccines are sub-unit protein vaccines consisting of L1 only virus like particles for HPV 16 and 18 only or HPV 6,11,16 and 18. These VLP vaccines are highly immunogenic generating high titres of type restricted neutralising antibody to L1 and robust immune memory.

It is generally considered that the >90% protection afforded by these vaccines against ano-genital disease associated with the vaccine HPV types is antibody mediated but at the present there is no immune correlate - no measurable antibody titre or other quantifiable immune response that correlates with protection. However the mechanism of viral entry may provide insight into antibody mediated protection. HPV infection of the epithelial basal cell has been shown to require epithelial micro-wounding and repair [47-49]. This is a slow, complex and multi-step process during which virus is accessible to serum neutralising antibody that enters via the exudate that accompanies wounding. Experimental evidence indicates that, at least for HPV 16 very low (pico-molar) antibody concentrations effectively neutralise suggesting that very little antibody will be protective [50].

## Competing interests

The author declares that they have no competing interests.
